# Acoustic Energy Release During the Laboratory Seismic Cycle: Insights on Laboratory Earthquake Precursors and Prediction

**DOI:** 10.1029/2019JB018975

**Published:** 2020-08-11

**Authors:** David C. Bolton, Srisharan Shreedharan, Jacques Rivière, Chris Marone

**Affiliations:** ^1^ Department of Geosciences Pennsylvania State University University Park PA USA; ^2^ Department of Engineering Science and Mechanics Pennsylvania State University University Park PA USA; ^3^ Dipartimento di Scienze della Terra La Sapienza Università di Roma Rome Italy

## Abstract

Machine learning can predict the timing and magnitude of laboratory earthquakes using statistics of acoustic emissions. The evolution of acoustic energy is critical for lab earthquake prediction; however, the connections between acoustic energy and fault zone processes leading to failure are poorly understood. Here, we document in detail the temporal evolution of acoustic energy during the laboratory seismic cycle. We report on friction experiments for a range of shearing velocities, normal stresses, and granular particle sizes. Acoustic emission data are recorded continuously throughout shear using broadband piezo‐ceramic sensors. The coseismic acoustic energy release scales directly with stress drop and is consistent with concepts of frictional contact mechanics and time‐dependent fault healing. Experiments conducted with larger grains (10.5 μm) show that the temporal evolution of acoustic energy scales directly with fault slip rate. In particular, the acoustic energy is low when the fault is locked and increases to a maximum during coseismic failure. Data from traditional slide‐hold‐slide friction tests confirm that acoustic energy release is closely linked to fault slip rate. Furthermore, variations in the true contact area of fault zone particles play a key role in the generation of acoustic energy. Our data show that acoustic radiation is related primarily to breaking/sliding of frictional contact junctions, which suggests that machine learning‐based laboratory earthquake prediction derives from frictional weakening processes that begin very early in the seismic cycle and well before macroscopic failure.

## Key Points

- Coseismic energy release during laboratory earthquakes scales directly with stress drop
- Acoustic energy radiated throughout the lab seismic cycle tacks fault slip rate and depends on contact junction size
- Acoustic energy from laboratory foreshocks and mainshocks derives from breaking and sliding of frictional contact junctions

## 1 Introduction

A key goal of earthquake forecasting has been to identify temporal variations in the physical properties within and around tectonic faults (so called seismic precursors). Yet, despite long‐term interest in this problem, there has been little progress in identifying systematic and reliable precursors to earthquake failure (Milne, 1899; Rikitake, 1968; Scholz et al., 1973). Several studies have documented the complexity of this problem and the lack of success in identifying robust earthquake precursors (e.g., Bakun et al., 2005). Nevertheless, temporal changes in wave speed and seismicity (e.g., foreshocks and preseismic slip) have been observed, in hindsight, prior to earthquake failure (Brenguier et al., 2008; Chen et al., 2010; Gulia et al., 2016; Gulia & Wiemer, 2019; Nanjo et al., 2012; Niu et al., 2008; Papadopoulos et al., 2010). Furthermore, recent studies based on machine learning (ML) show that the timing, instantaneous shear stress, and, in some cases, the magnitude of laboratory earthquakes can be predicted using statistics of the continuous acoustic emission (AE) signal emanating from the fault zone (Hulbert et al., 2019; Lubbers et al., 2018; Rouet‐Leduc et al., 2017, 2018). The lab‐based studies are simplified analogs to tectonic faulting, but there are enough similarities between lab events and earthquakes (e.g., Brace & Byerlee, 1966; Scholz, 1968, 2015) to warrant further study.

**Figure 1:**
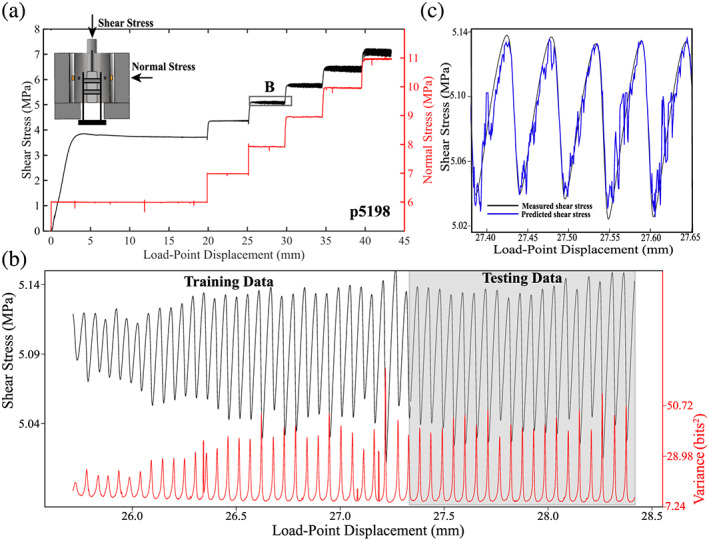
(a) Data for one complete experiment (p5198) showing measured stresses as a function of load‐point displacement. Inset in (a) shows double‐direct shear configuration with acoustic sensors (orange squares) and onboard displacement transducer. Shear and normal forces are measured with strain gauge load cells mounted in series with the vertical and horizontal rams, respectively. Horizontal and vertical displacements are measured with direct current displacement transformers and are referenced to the loading frame. (b) Zoom of shear stress and acoustic energy during a series of lab earthquakes. Note the systematic evolution of acoustic variance throughout the seismic cycle. For the ML analysis (see Hulbert et al., 2019), we use the first 60% of the data for training and the remaining 40% for testing. (c) Comparison of measured and predicted shear stress (*r* ^2^ = 0.87) using ML.

Previous ML works demonstrate that the variance of the acoustic signal, which is a proxy for the average acoustic energy per unit time, is a key parameter for successful lab earthquake prediction (Figure 1; Hulbert et al., 2019; Rouet‐Leduc et al., 2018). Of the ~100 statistical features tested, AE signal variance was found to be the most important predictor of shear stress and fault failure time (Rouet‐Leduc et al., 2017, 2018). Despite these observations, it is unclear how AE signal variance is connected to the physical state of the fault. In particular, the mechanisms of AE radiation and their evolution during the seismic cycle, which provides the physical basis for lab earthquake prediction, are unknown (Figures 1b and 1c). Answers to such questions will help illuminate the mechanisms behind seismic precursors and, thus, improve our physical understanding of ML‐based predictions of laboratory earthquakes.

There are strong parallels between ML‐based lab earthquake prediction and previous laboratory studies that have focused on the spatiotemporal evolution of seismic precursors to laboratory earthquakes (Bolton et al., 2019; Goebel et al., 2013, 2015; Johnson et al., 2013; Kaproth & Marone, 2013; Latour et al., 2011; Latour, Schubnel, et al., 2013; Latour, Voisin, et al., 2013; Renard et al., 2017; Rivière et al., 2018; Rubinstein et al., 2007, 2009; Scuderi et al., 2016; Shreedharan et al., 2020; Tinti et al., 2016; Weeks et al., 1978). In particular, passive acoustic measurements show that there are pervasive foreshocks that precede most laboratory earthquakes. Both the frequency and magnitude of the foreshocks increase before the main slip event, and as a result, the Gutenberg‐Richter b‐value decreases systematically before failure (Goebel et al., 2013, 2015; Jiang et al., 2017; Johnson et al., 2013; Lei & Ma, 2014; Lockner et al., 1991; Main et al., 1989; McLaskey & Lockner, 2014; Ohnaka & Mogi, 1982; Rivière et al., 2018; Sammonds et al., 1992; Scholz, 1968; Thompson et al., 2005, 2009; Weeks et al., 1978). In addition, active source measurements show clear precursory changes in fault zone properties, such as elastic wave speed prior to failure (Crampin et al., 1984; Gupta, 1973; Kaproth & Marone, 2013; Lockner et al., 1977; Niu et al., 2008; Scuderi et al., 2016; Shreedharan et al., 2019, 2020; Tinti et al., 2016; Whitcomb et al., 1973). Previous studies have demonstrated that microfactures nucleate and coalesce prior to rock failure (Brace & Bombolakis, 1963; Paterson & Wong, 2005; Scholz, 1968; Tapponnier & Brace, 1976). In addition, recent experiments have illuminated this process in higher detail using X‐ray microtomography (Renard et al., 2017, 2018). Thus, numerous observations indicate that laboratory earthquakes are preceded by a preparation phase that involves physical changes in the fault zone; however, the underlying mechanisms and the physical processes that cause precursors and allow prediction are poorly understood.

Here, we report on a suite of friction experiments to illuminate the physical mechanisms that control the evolution and magnitude of acoustic energy released during frictional sliding. We study both stable frictional sliding and unstable stick‐slip sliding. Stick‐slip experiments were conducted over a range of boundary conditions to explore the physical properties that dictate the evolution of the acoustic energy. We augment data from frictional sliding experiments with slide‐hold‐slide (SHS) frictional tests in order to show that acoustic radiation during the lab seismic cycle may be primarily controlled by processes at frictional contact junctions.

## 2 Methods

We report on a suite of friction experiments on quartz powder conducted in a double‐direct shear (DDS) configuration (inset to Figure 1a). In this configuration, two layers of fault gouge are sheared at constant fault normal stress between rough, steel forcing blocks (e.g., Frye & Marone, 2002). Our experiments are conducted at constant shear velocity, which involves controlling the velocity of the fault zone boundary (Figure 1a) with a fast‐acting servo‐controlled ram. We varied normal stresses from 6–11 MPa, shearing velocities from 2–60 μm/s, and median grain sizes from 1.7–10.5 μm (Table 1). Forces and displacements were measured continuously at 1 kHz with strain‐gauge load cells and direct current displacement transformers (DCDT). Fault slip was measured with a DCDT attached directly to the center forcing block of the DDS assembly and referenced to the bottom of the load frame (Leeman et al., 2018; Figure 1a). Fault slip velocity is computed using a moving window approach on the data recorded by the DCDT mounted directly to the center block. To eliminate variation between experiments due to humidity (e.g., Frye & Marone, 2002), all tests were conducted at 100% relative humidity. Prior to each experiment, both layers were placed inside a plastic bag with a 1:2 sodium carbonate and water solution and allowed to sit overnight for 12–15 hr. To ensure constant relative humidity throughout the experiment, humid air was blown into a plastic chamber around the loading blocks.

**Table 1:** List of Experiments and Boundary Conditions

Experiment	Normal stress (MPa)	Drive velocity (μm/s)	Median grain size (μm)
p5198	6–11	10	10.5
p5201	9	2–60	10.5
p5263	10	10	10.5
p5264	10	10	4.67
p5273	10	2–60	10.5
p5293	10	10	1.67
p5317	9	2–60	10.5
p5348	9	2–60	10.5

Gouge layers were constructed using cellophane tape and a leveling jig (e.g., Anthony & Marone, 2005; Karner & Marone, 1998). In addition, side plates were mounted between the side blocks and center block to limit extrusion of material along those edges. After the sample was humidified overnight, the DDS assembly was placed inside the load frame, and a normal force was applied perpendicular to the sample. The sample was then left to compact for 30–40 minutes until the layer thickness reached a steady‐state value. Once the sample reached a constant layer thickness, the center block was driven down to induce a prescribed shear velocity at the layer boundary.

We observe a spectrum of slip behaviors from stable sliding to unstable stick‐slip instabilities, which are the lab equivalent of earthquakes. For stick‐slip sliding, we observe a continuum of behaviors ranging from slow slip to fast, dynamic slip events (Leeman et al., 2015, 2016, 2018; Scholz et al., 1972; Scuderi et al., 2016). To produce a spectrum of slip behaviors, we modulate the loading stiffness *k*, by placing an acrylic spring in series with the vertical ram, such that our effective loading stiffness is equal to the critical frictional weakening rate, *k*_*c*_ (Gu et al., 1984; Leeman et al., 2015, 2016).

We measured AEs continuously throughout the experiment using broadband (~0.0001–2 MHz) lead‐zirconate‐titanate piezoceramic sensors (Rivière et al., 2018; supporting information Figure S4). The piezoceramic sensors (12.7 mm diameter; 4 mm thick) are embedded inside steel blocks and placed ~18 mm from the fault zone (Bolton et al., 2019; Rivière et al., 2018). Acoustic data were recorded continuously throughout the experiment at 4 MHz using a 15‐bit Verasonics data acquisition system. Our experiments include data from two sensors. We conducted many calibration experiments and tests and found only minor differences between the sensors (Rivière et al., 2018). Thus, we focus here on data from one sensor.

The acoustic variance, A_V_, (Equation 1) is calculated as

(1) $$\;\mathrm{Av}=\frac{1}{N}\;\sum_{i=1}^{N}{\left(a_{i}-\bar{a}\right)}^{2},$$

where *a*_*i*_ is the amplitude of the time series signal at index *i*, *N* is the number of data points considered in a moving window, and $\bar{a}$ is the mean value in the window of size *N* (Hulbert et al., 2019; Rouet‐Leduc et al., 2017, 2018). In this work, we use the terms variance and acoustic energy interchangeably since variance is proportional to the acoustic energy release. We use a moving window on the acoustic time series data to compute the acoustic variance. The size of the moving window is selected such that it is less than or equal to 10% of the recurrence interval. This approach ensures that the windows are small relative to the recurrence interval of the seismic cycle. Each moving window overlaps the previous window by 90%, and we use a center‐based time stamp for each window (i.e., it is therefore forward looking by a half a window length).

## 3 Results

**Figure 2:**
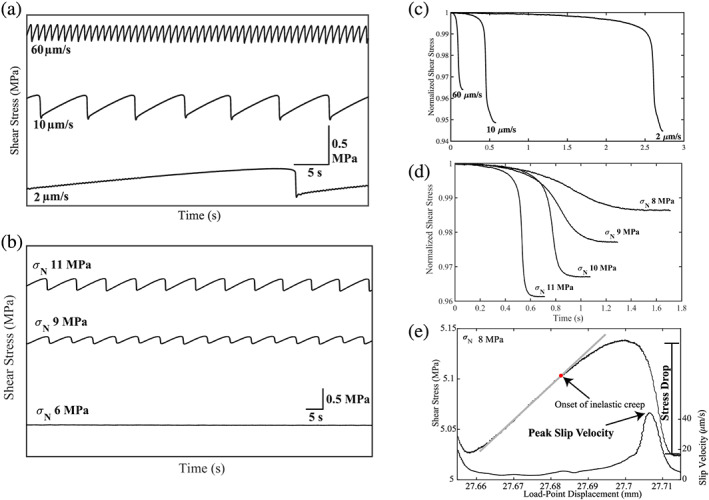
(a, b) Shear stress plotted as a function of time for data at different shear velocities and normal stresses (a, 2–60 μm/s; b, 6–11 MPa). Note that the lab seismic cycle changes systematically with shear velocity and normal stress. The stress drop during failure events decreases as fault normal stress decreases, and sliding becomes stable at the lowest normal stress. (c) Shear stress normalized by the peak value prior to failure is plotted as a function of time for three different driving velocities. Note that stress drop scales inversely with shear velocity. (d) Normalized shear stress during failure events at four normal stresses. Slip duration decreases and stress drop increases as normal load increases. (e) Shear stress and slip velocity as a function of load‐point displacement for one seismic cycle. Gray line shows elastic loading when the fault is locked. The onset of fault slip (inelastic creep) is marked with the red dot. Note that the onset of inelastic creep varies with normal stress and shear velocity. The fault reaches its peak slip velocity during coseismic failure. Stress drop is calculated as the difference between the peak shear stress and the minimum shear stress.

We conducted experiments over a range of boundary conditions (Table 1). All stick‐slip experiments start with a period of stable sliding followed by emergent quasiperiodic unstable slow slip (Figure 1a). As shearing continued, the magnitude of the stick‐slip events typically reached a steady state. We systematically modify the characteristics of the stick‐slip events by changing the loading rate, normal stress, and grain size (Figure 2). For example, in Experiment p5198, we varied normal stress and observed a spectrum of slip behaviors (Figures 2b and 2d). At a normal stress of 6 MPa, the slip events contain very small stress drops; however, after increasing the normal load to 7 MPa (not shown), the magnitude of the stress drop increases and eventually reaches a steady‐state value (see data at 8–11 MPa in Figures 2b and 2d). For Experiment p5201, we systematically modulate the characteristics of the slip cycles by changing the shear velocity from 2 to 60 μm/s (Figures 2a and 2c). At low shear velocities, slip events have long recurrence intervals and large stress drops, while at high shear velocities, the recurrence intervals are shorter, and stress drops are smaller (Figures 2a and 2c). The early stage of each loading cycle is characterized by linear‐elastic loading followed by the onset of inelastic creep (Figure 2e). During inelastic loading, the fault slip velocity begins to increase, and it reaches a peak during the coseismic slip phase.

### 3.1 Acoustic Energy

**Figure 3:**
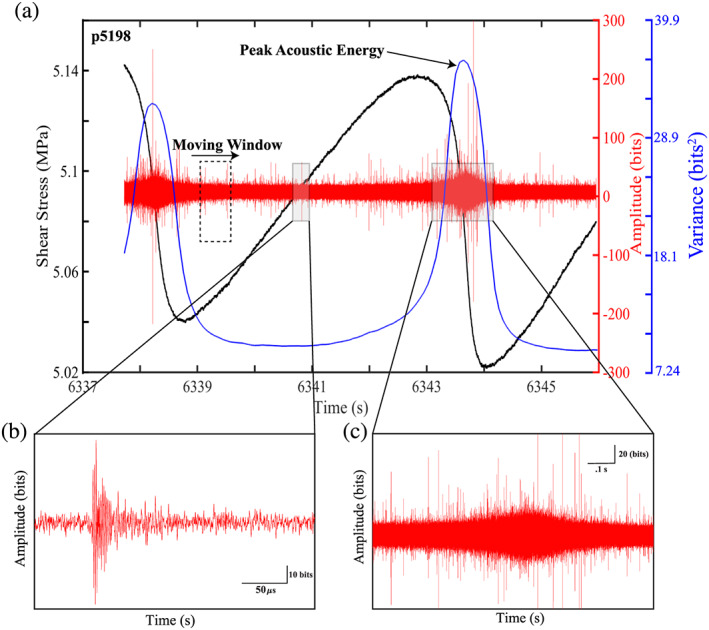
(a) Shear stress, acoustic amplitude, and acoustic variance plotted as a function of time for one seismic cycle. The dashed rectangle shows our moving window (0.636 s) used to compute the acoustic variance. At this scale acoustic data look like noise; however, the signal is composed of individual AEs (some identifiable as small spikes) that grow in size and number as failure approaches (see b). The acoustic variance first decays following a failure event, reaches a minimum during the interseismic period, and finally begins to increase prior to failure. (b) Zoom of an AE that nucleated during the interseismic period. (c) Zoom of the acoustic signal during coseismic failure. Note the broad, low amplitude nature of the envelope with superimposed high‐frequency AEs.

In Figure 3, we show an example of the AE data for one of the hundreds of failure events analyzed. Note that these data are from a portion of Experiment p5198 at 8 MPa and contain slow‐slip events. During the interseismic period, the acoustic time series signal is composed mainly of what looks like noise with a few small discrete AEs (small spikes in the signal; Figure 3b). However, one can observe that the number and size of the AEs increase as failure approaches. This is also observed in the temporal trends of the acoustic energy (Figure 3). In addition to the interseismic trends, the acoustic data associated with the coseismic slip phase have a unique character. In particular, the envelope of the raw acoustic signal has a broad‐low amplitude signature during the coseismic slip phase (Figure 3c). In addition, there are many high‐frequency AEs, like the one shown in Figure 3b, that occur throughout the coseismic slip phase.

The radiated acoustic energy evolves systematically during the slip cycle (Figure 3). Here, a window length of 0.636 s is used to compute the acoustic variance, which is time stamped to the center of the window (Figure 3). After a failure event, the acoustic variance first decays and reaches a minimum value. It then increases gradually and reaches a peak value during failure (Figures 1 and 3). Note that the increase in acoustic variance begins prior to coseismic failure (Figure 3). To fully understand the characteristics of the acoustic energy, we focus on the details of the temporal behavior of the acoustic energy as well as other systematics such as the scaling relationship between the cumulative acoustic energy radiated during coseismic slip, stress drop, and peak slip velocity.

### 3.2 The Influence of Normal Stress and Shear Velocity on Acoustic Energy

**Figure 4:**
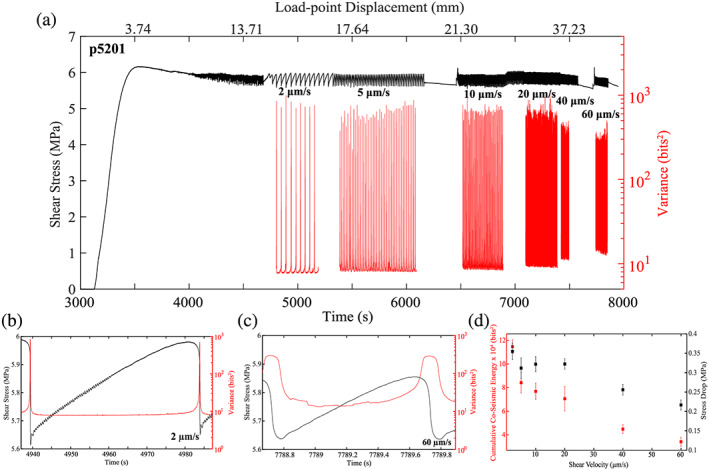
(a) Shear stress and acoustic variance plotted as a function of time and load‐point displacement for one complete experiment (p5201) with detail at (b) 2 μm/s and (c) 60 μm/s. Note, the variance in (a) is a discrete time series signal computed at all times throughout the seismic cycle. When plotted on the same scale, the acoustic variance time series shows distinct differences as a function of velocity. At low shear velocity, the acoustic variance stays low for most of the seismic cycle and only begins to increase once the fault has reached its peak strength. In contrast, at high drive velocities, the acoustic variance decays, reaches a minimum, and begins to increase before the fault reaches its peak stress. (d) Average cumulative acoustic energy and stress drop plotted as a function of shear velocity. The cumulative acoustic energy is computed from the variance time series data in (a). Variance is integrated from peak shear stress to minimum shear stress for each slip cycle shown in (a). Square symbols represent mean values, and error bars represent one standard deviation. Cumulative acoustic energy scales directly with stress drop and inversely with shear velocity.

Our results demonstrate that shear velocity has a significant influence on the temporal evolution and magnitude of acoustically radiated energy during the lab seismic cycle (Figure 4). For Experiment p5201, the normal load was held constant at 9 MPa, while the shear velocity was varied from 2–60 μm/s. For each test, the initial shear velocity was 10 μm/s. After shearing ~14 mm, the shear velocity was decreased to 2 μm/s and subsequently increased from 2–60 μm/s after shearing between 1 and 8 mm at each shear velocity. Shear stress and acoustic variance are plotted as functions of time and load‐point displacement (top) in Figure 4. For Experiment p5201, a constant time window of 0.1 s is used to compute the acoustic variance. We plot shear stress and acoustic variance as functions of time for a representative stick‐slip cycle at 2 and 60 μm/s, respectively, in Figures 4b and 4c. Plotting the acoustic variance on the same scale reveals distinct differences in the temporal variations in acoustic variance throughout the stick‐slip cycle. In particular, at 60 μm/s, the acoustic variance first decreases, reaches a minimum, and then begins to increase prior to failure. At 2 μm/s, the acoustic variance decreases, reaches a minimum, and remains there throughout the interseismic period before it finally increases just before failure. In addition to the temporal trends, we plot the cumulative acoustic energy (i.e., variance) during coseismic rupture and stress drop as a function of shear velocity in Figure 4d. The cumulative acoustic energy is computed from peak shear stress to minimum shear stress for the variance data shown in Figure 4a. We focus on cumulative acoustic energy rather than the peak energy to avoid artifacts of different window lengths (Figure S2). The data show that the cumulative acoustic energy radiated during coseismic failure scales inversely with shear velocity and linearly with stress drop (Figures 4a and 4d). In addition to the temporal trends in acoustic variance, the minimum acoustic variance reached during the interseismic period varies systematically with shear velocity. At 2 μm/s, the minimum acoustic variance is slightly lower (~10 bits^2^) compared to the minimum acoustic variance at 60 μm/s (~20 bits^2^).

It is important to note that we use a constant time window of 0.1 s to compute the acoustic variance in Figure 4. The length of the moving window corresponds to 10% of the recurrence interval for data at 60 μm/s, and since recurrence interval scales inversely with shear velocity, this ensures that all moving windows are less than or equal to 10% of the recurrence interval. Since windows are constant in time, the amount of slip displacement covered by each moving window increases with shear velocity. We demonstrate that the acoustic variance is independent of slip displacement by using different windowing techniques (see Supporting Information S1) and analyzing acoustic data during stable frictional sliding experiments (Figures S1 and S2). In particular, we compute acoustic variance using a moving window that is constant in slip displacement (Figures S1a and S2). Similar to Figure 4, the data show that more energy is released at higher shear velocities (Figure S1a). However, since we use a constant displacement window in Figure S1 and acoustic data are recorded at a constant sampling frequency in time, the number of data points (*N*) considered in each moving window changes systematically with shear velocity. In other words, the window size (*N* in Equation 1) decreases with increasing shear velocity. To circumvent this issue, we decimated the acoustic data such that the number of data points is the same for each moving window. Again, the data show an increase in energy release with increasing shear velocity, and the absolute values of variance do not change for the decimated case (Figures S1a and S1b). Similarly, data from stick‐slip experiments (e.g., p5201) demonstrate that the interseismic changes in energy are independent of window length and slip displacement (Figure S2). However, acoustic data associated with the coseismic slip phase are affected by the window length (Figure S2). As mentioned above, we avoid the issue of window size during the coseismic slip phase by reporting on the cumulative energy released rather than peak energy. In conclusion, the results shown in Figure 4 are independent of slip displacement, and the interseismic trends are independent of the window size (see Supporting Information S1).

**Figure 5:**
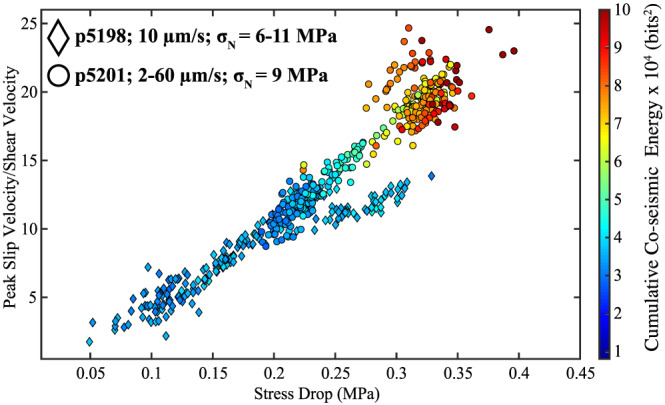
Normalized peak slip velocity during failure as a function of stress drop for all events in two experiments. Symbols are color coded according to the cumulative acoustic energy. Note the strong correlation between peak slip velocity, stress drop, and cumulative acoustic variance radiated from the fault during failure.

Our data show a robust relationship between the stress drop of the stick‐slip event and the amount of acoustic energy radiated from the fault (Figure 5). In Figure 5, we show results from two experiments, p5198 (diamond symbols) and p5201 (circle symbols). For these experiments, we systematically change the stress drop of the slip events by changing the normal stress and shear velocity (see Figure 2). The relationship between stress drop and slip velocity as functions of normal stress and shear velocity is consistent with previous works (Leeman et al., 2016, 2018; Scuderi et al., 2016). Data from Experiment p5201 are plotted in the upper right corner of Figure 5, while data from p5198 are plotted in the lower left corner of Figure 5. These data show that fast laboratory earthquakes release greater amounts of acoustic energy during coseismic failure compared to slow slip events.

**Figure 6:**
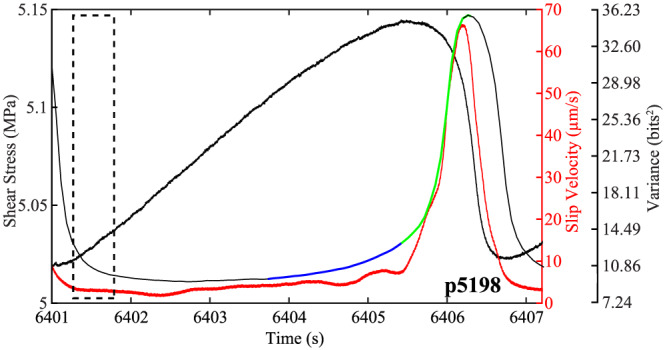
Shear stress, acoustic variance, and slip velocity as a function of time for one seismic cycle in Experiment p5198 (8 MPa normal stress). Dashed rectangle shows the moving window used to compute the acoustic variance. Initially, the fault is locked, with near‐zero slip velocity. The fault begins to unlock about half way through the cycle, and the fault slip rate increases dramatically prior to failure. The acoustic variance mimics the slip velocity and reaches a peak during coseismic failure. Acoustic variance is color coded based on the following: Black to blue shows the onset of inelastic creep, blue to green coincides with the peak shear stress, and green to black corresponds to the peak slip velocity.

To illuminate the mechanisms controlling the temporal evolution of acoustic energy throughout the interseismic period, we plot the acoustic variance, shear stress, and slip velocity for one seismic cycle in Figure 6. After the failure event, the acoustic variance begins to decay and finally reaches a minimum at around 6402.5 s. Interestingly, at this same time, the slip velocity is also at a minimum. Following the minimum, the acoustic variance begins to increase and reaches a peak during the coseismic slip phase. Again, at approximately the same time that the acoustic variance begins to increase, the fault begins to unlock and accelerate forward. Because the amount of inelastic creep varies systematically with normal stress and shear velocity, we further probe the evolution of acoustic variance during the interseismic period by showing the effects of normal stress and shear velocity on the interseismic changes in acoustic variance.

**Figure 7:**
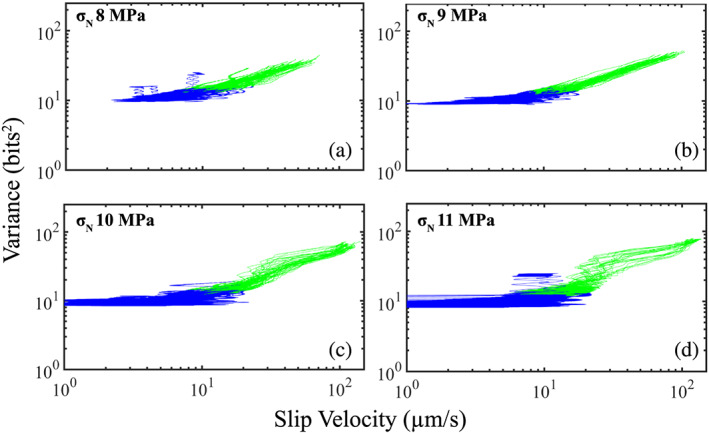
Acoustic variance as a function of slip velocity plotted for four different normal stresses from Experiment p5198. Plots show data from multiple slip cycles at each load (see Figure 1). For each slip cycle, we plot data from the onset of inelastic creep until peak‐slip velocity. Blue shows data from the onset of inelastic creep until peak shear stress. Green shows data from peak shear stress until peak slip velocity (see Figures 2e and 6). (a, b) At low normal loads (8–9 MPa), the acoustic variance increases with slip velocity during the interseismic period (blue data). Also note that the acoustic variance increases only as the fault reaches a slip rate of ~10 μm/s. At higher normal loads (10–11 MPa), the fault slip rate is <10 μm/s for most of the interseismic period, and the acoustic variance only increases during the latter stages (green) of the seismic cycle.

**Figure 8:**
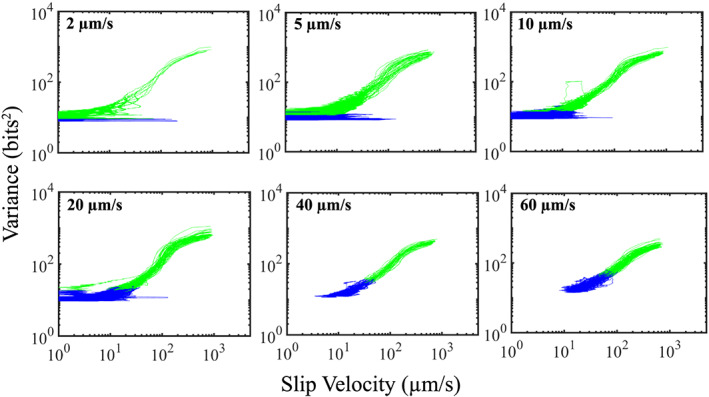
Acoustic variance as a function of slip velocity for data at six different shear velocities from Experiment p5201 (same color coding as Figure 7). At low shear velocities (2–5 μm/s), the acoustic variance does not increase during the interseismic period (e.g., blue data). In contrast, at high shear velocities (≥20 μm/s), the acoustic variance increases systematically with slip velocity during the interseismic period.

For fault zones composed of large grain sizes (10.5 μm), our data show that the acoustic variance increases when the fault unlocks and begins to accelerate (Figure 6). Therefore, for each stick‐slip cycle, we focus our analysis from the onset of inelastic creep until the fault has reached its peak slip velocity during coseismic failure. In Figures 7 and 8, we highlight these segments of the seismic cycle with blue and green colors. Data in blue are from the onset of inelastic creep until peak shear stress, and those in green are from the peak shear stress until the peak slip velocity (see Figure 6). We plot acoustic variance as a function of slip velocity from multiple slip cycles (see Figure 2) at four different normal stresses in Figure 7. The data show that the slip rate of the fault is higher at the onset of creep for lower normal stresses. That is, the fault slip rate is less than 1 μm/s at the onset of creep for data at 10–11 MPa, but for data at 8–9 MPa, the slip rate is faster at the onset of creep (between 1 and 10 μm/s). The differences in minimum slip rate as a function of normal stress have a direct consequence on whether or not the acoustic variance begins to increase or remain at steady‐state value. For data at 8–9 MPa, the acoustic variance begins to increase once the fault unlocks. However, for data at 10–11 MPa, the acoustic variance remains low even when the fault begins to creep and only increases when the fault is near its peak shear stress (the transition from blue to green). In general, it seems that the fault slip rate must be ~10 μm/s before the acoustic energy begins to increase. For data at 10–11 MPa, the fault only reaches this slip rate near the onset of peak shear stress, while at 8–9 MPa, the fault reaches this slip velocity earlier in its seismic cycle.

In Figure 8, we show how shear velocity influences the relationship between slip velocity and acoustic variance. Similar to the data at high normal stresses, the acoustic variance at low shear velocities (2–10 μm/s) does not increase prior to the peak shear stress (i.e., the transition from blue to green). However, at higher shear velocities (>20 μm/s), the acoustic variance begins to increase prior to reaching peak stress. In addition, note that the acoustic variance does not begin to increase until the fault has reached a slip velocity of ~10 μm/s. Furthermore, since the fault stays locked longer at low shear velocities, it fails to reach this slip velocity during the interseismic period. However, at higher shear velocities, the fault reaches this slip velocity early on in its seismic cycle and reaches a higher slip velocity upon peak shear stress as the background loading rate increases. For example, the slip rate of the fault at the onset of creep is around 10 μm/s for data at 40 and 60 μm/s, and the fault reaches a slip velocity of ~40–60 μm/s at peak shear stress. In contrast, the slip rate of the fault at the onset of creep at 10 μm/s is ≤1 μm/s, and the fault reaches a slip velocity of only ~10 μm/s at peak shear stress.

### 3.3 SHS Tests

**Figure 9:**
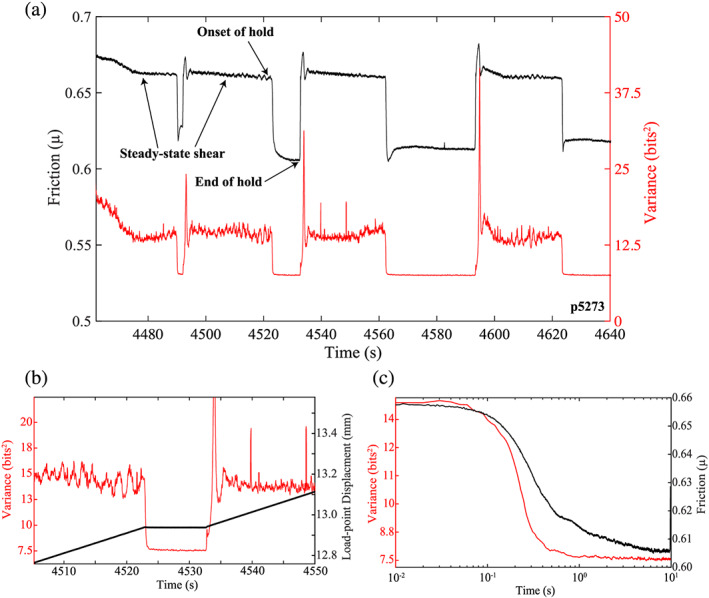
(a) Friction and acoustic variance plotted as a function of time for a series of SHS tests for Experiment p5273. Here, we use a 0.1 s window to compute the acoustic variance. Acoustic variance remains at a steady‐state value during sliding and decreases rapidly at the start of a hold. Upon reshear, the variance increases, reaches a peak, and decays back to the steady‐state value. (b) Acoustic variance and load‐point displacement as a function of time. Note that acoustic variance tracks fault slip‐rate. (c) Acoustic variance and friction plotted as function of log time for a 10 s hold (see a). Both the acoustic variance and friction decay rapidly at the onset of the hold. However, the acoustic variance drops to a steady‐state value, whereas friction continues to decrease throughout the hold.

To further verify that the acoustic variance is linked to fault slip rate, we conducted conventional SHS friction tests. These SHS tests were also conducted to help illuminate the relationship between frictional restrengthening processes and the generation of acoustic energy. In conventional SHS tests, the fault is initially sheared at a constant displacement rate, followed by a pause in shearing, and is finally resheared at the same displacement rate prior to the hold (Dieterich, 1972, 1978; Marone, 1998). During a typical SHS test, friction first decays during the hold and then reaches a maximum value upon reshear (Figure 9a). Our data show that the acoustic variance tracks the frictional evolution throughout the entire SHS test. Once the fault stops sliding, the acoustic variance decreases significantly, followed by a gradual decay to a steady‐state value (Figures 9b and 9c). Upon reshear, the acoustic variance begins to increase and reaches a maximum followed by a decay to a steady‐state value. These data corroborate our findings above and demonstrate that for fault zones composed of large particles, the acoustic variance tracks fault slip rate.

### 3.4 The Influence of Grain Size on Acoustic Energy

**Figure 10:**
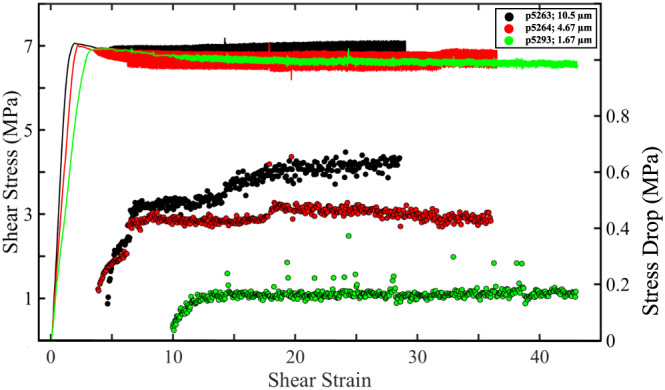
Shear stress and stress drop as a function of shear strain for experiments conducted with different median grain sizes. Note that stress drop increases during the initial part of each experiment and reaches a steady state for which larger grains produce bigger events.

**Figure 11:**
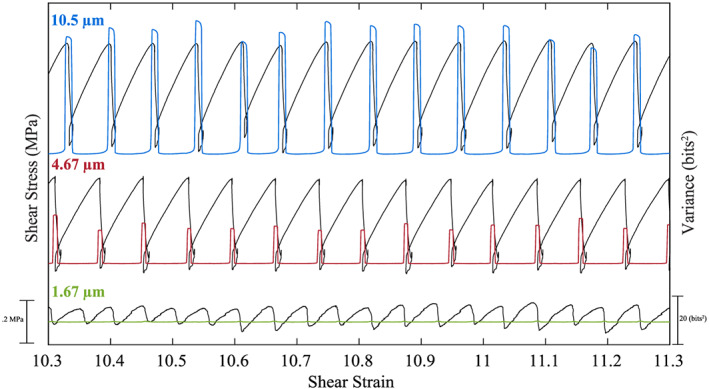
Shear stress and acoustic variance versus shear strain for fault gouge composed of different median grain sizes. Plots are offset vertically for clarity. Fault zones composed of larger grains produce larger stress drops, have longer recurrence intervals, and radiate more energy during coseismic failure.

**Figure 12:**
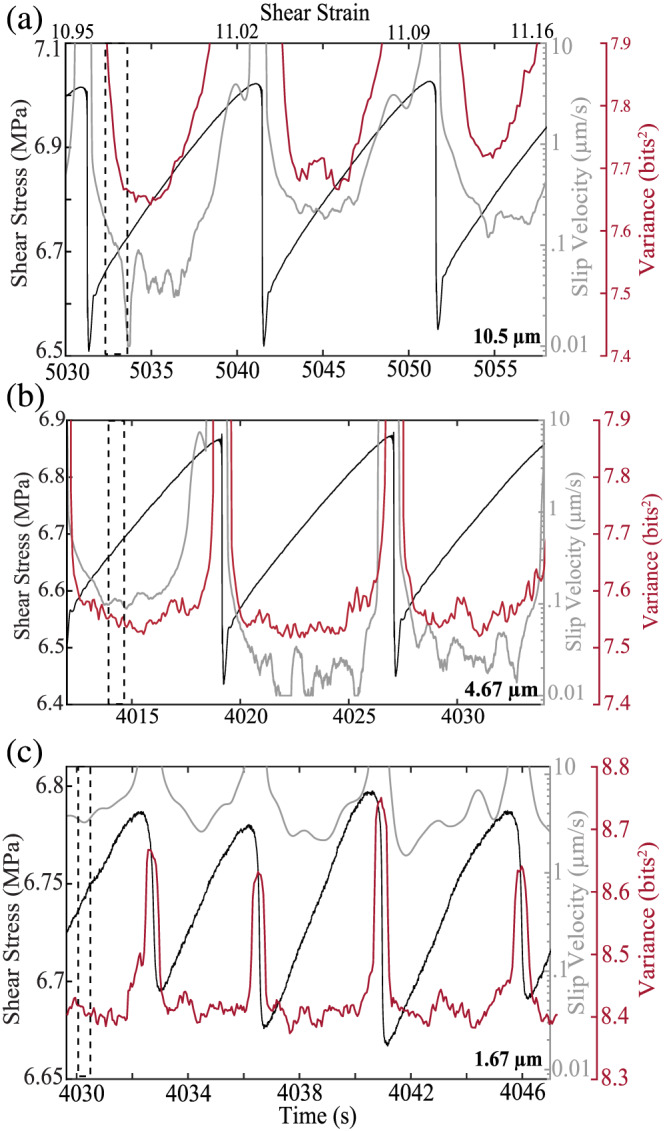
(a–c) Zoom of each experiment shown in Figure 11. Note the acoustic variance range is the same for each plot. The acoustic variance begins to increase later in the seismic cycle for fault zones composed of smaller grains.

We varied fault zone grain size in order to study the impact of frictional contact junction size on stick‐slip dynamics and acoustic energy (Figures 10, 11, 12). For each experiment in Figure 10, we change the median grain size of the fault gouge while maintaining a constant normal load, shear velocity, and initial layer thickness (Table 1). Furthermore, each material consists of monodispersed particles with a similar, narrow, size range. We plot shear stress and stress drop as a function of shear strain in Figure 10. We compute the instantaneous shear strain by integrating the load‐point displacement data normalized by the layer thickness (Scott et al., 1994). Despite the fact that our range of median grain sizes is less than an order of magnitude, the character of the slip cycles varies significantly (Figure 10). Our data show distinct differences in stick‐slip properties as a function of median grain size. In particular, fault strength, recurrence interval, and stress drop increase as a function of the median grain size (Figure 10).

Fault gouge grain size also has a significant impact on radiated acoustic energy (Figure 11 and 12). Our data show that the peak acoustic variance scales systematically with grain size and stress drop (Figure 11). We show the temporal evolution of stress, slip velocity, and acoustic variance for multiple seismic cycles in Figures 12a–12c. Fault zones composed of larger particles (median diameter of 10.5 μm) show a decrease in acoustic variance following failure and then an increase prior to failure (Figure 12a). As noted above, this temporal behavior tracks fault slip velocity. However, for grain sizes smaller than 10.5 μm, these temporal trends seem to diminish (Figures 12b and 12c). That is, for small particles (median sizes of 1.67 and 4.67), the increase in acoustic variance prior to failure is significantly reduced. Moreover, for the smallest grains, the acoustic variance does not increase prior to failure, despite the fact that the fault slip rate is rather high during the interseismic period (~10 μm/s). Rather, the acoustic variance seems to fluctuate around a mean value before reaching its peak during coseismic slip (Figure 12c).

## 4 Discussion

### 4.1 The Effects of Normal Stress and Shearing Velocity on Acoustic Energy

Previous ML studies (e.g., Rouet‐Leduc et al., 2017) have found that the acoustic variance (energy) is one of the main features that enable laboratory earthquake prediction. The temporal evolution in acoustic energy is what ultimately enables certain aspects of laboratory earthquakes to be predicted. However, the physics that control the release of acoustic energy prior to failure has been poorly understood. In this work, by focusing on the physical parameters that control acoustic energy release throughout the seismic cycle, we are able to offer a physical explanation behind the ML‐based predications of laboratory earthquakes and their associated precursors.

We carried out a suite of experiments to better understand the physical mechanisms that control the magnitude and temporal evolution of acoustic energy release throughout the laboratory seismic cycle. We find a robust relationship between the cumulative acoustic energy released during coseismic slip and the stress drop of the slip event (Figures 5). This relationship exists over a range of normal stresses, shear velocities, grain sizes, and over a spectrum of slip events ranging from slow to fast dynamic events. The total amount of energy released during coseismic rupture is a function of the experimental boundary conditions. For each experiment, we directly control the amount of energy stored within the fault zone by systematically changing the normal stress, shearing velocity, and grain size. At high normal loads and low shearing velocities, the fault stays locked longer during the interseismic period, which allows more frictional healing to take place. Similarly, for a constant normal load and shearing velocity, more frictional healing takes place during the interseismic period for fault zones composed of larger particle sizes. This increase in frictional strength allows the fault zone to accumulate more elastic‐strain energy during the interseismic period. However, once the fault begins to unlock and creep, a portion of this stored elastic‐strain energy is released through acoustic waves, while part of the remaining acoustic energy is released during coseismic failure. Our data show that the total acoustic energy released during coseismic rupture scales with the size of the stress drop (Figures 4 and 5). Our data are consistent with field observations that show a systematic relationship between energy, seismic moment, magnitude, and duration (Ide et al., 2007; Kanamori et al., 1993; Vassiliou & Kanamori, 1982). This suggests a simple micromechanical model in which larger magnitude slip events experience more interseismic frictional healing, and as a result of this increase in strength, they release more acoustic/seismic energy during coseismic failure when grain contacts are destroyed.

Our data show that the lowest level of acoustic energy release during the lab seismic scales systematically with shear velocity (Figure 4). The minimum energy shown in Figure 4 occurs approximately where the inelastic loading phase begins and, thus, represents the point at which grain contact junctions begin to slip and break. However, it is important to point out that AEs do occur during the linear‐elastic loading phase (Figure 3). This suggests that grain contact junctions have already started to slide and break during this phase. Previous works have demonstrated that there is a net increase in the number of contacts and contact area during the linear‐elastic loading phase (Shreedharan et al., 2019). However, since both the slip velocity and acoustic energy are low during the linear‐elastic loading phase, we hypothesize that the total number of contact junctions breaking is low, and healing mechanisms dominate. In contrast, once the fault begins to unlock and creep (i.e., slip velocity > 0), the total number of contact junctions breaking increases significantly and results in a subsequent increase in energy radiation. This idea is consistent with the data presented in Figure 4 and with physical models of frictional contact and contact aging (e.g., Li et al., 2011; Shreedharan et al., 2019). That is, young grain contacts are smaller, weaker, and have less time to heal at higher slip rates, which allows for more contacts to break prior to failure at faster slip rates. The temporal trends in acoustic energy further verify this hypothesis (Figures 7 and 8). That is, the temporal changes in acoustic energy release during the interseismic period is greater for higher shear velocities (Figures 4b, 4c, 7, and 8). More specifically, at high shear velocities/low normal stresses, the slip rate of the fault is much higher during the interseismic period, which enhances destruction of grain contact junctions. If the acoustic energy is related to the slipping/breaking of contact junctions, we should expect a higher rate of acoustic energy release to occur with higher slip rates. In contrast, at low shear velocities/high normal stresses, the fault stays locked longer, and when it does unlock, the fault slip rate is much lower. This process results in more frictional healing, and as a result, less contacts are slipping and breaking, which reduces the rate of acoustic energy released during the interseismic period. Therefore, our data demonstrate that the magnitude and temporal changes in acoustic energy release are controlled by fault slip velocity. Our results are consistent with previous laboratory works that have shown higher amounts of AE activity with increasing strain rate/shearing velocity (Jiang et al., 2017; McLaskey & Lockner, 2014; Ojala et al., 2004; Yabe, 2002). These findings could have important implications for microseismic activity and precursors to frictional failure (e.g., Brodsky, 2019; Gulia & Wiemer, 2019; Ross et al., 2019; Trugman & Ross, 2019). Our data suggest that there could be an insignificant amount of seismic activity released prior to larger earthquakes if the fault stays locked up and the minimum slip rate attained by the fault is low. In contrast, if the fault does unlock and begins accelerating, there could be a substantial increase in seismic activity preceding failure. Furthermore, our data demonstrates that the acoustic energy radiating from the fault zone is fundamentally linked to the fault slip rate. This is consistent with recent observations of deep low‐frequency earthquakes in Mexico where the maximum *S* wave amplitude of low‐frequency earthquakes qualitatively tracks fault slip rate constrained by geodesy (see Figure 1 from Frank & Brodsky, 2019). Therefore, our results could be particularly useful to help us understand the physics of slow earthquakes.

It is important to note that once the fault unlocks and the onset of inelastic loading occurs, both shear stress and slip velocity begin to increase. Therefore, one could equally argue that acoustic energy tracks shear stress during inelastic loading, which has been shown in previous works (Passelègue et al., 2017). However, our data clearly show that slip velocity is the main parameter that controls acoustic energy release and not shear stress. To demonstrate that shear stress is not the dominant parameter, we plot data from the onset of inelastic creep until peak stress (i.e., blue data in Figures 7 and 8) in Figure S3. Data from Experiment p5198 show that the amount of energy released prior to failure scales inversely with friction. If acoustic energy tracked shear stress, we should expect to see more energy released for higher values of friction. However, our data show that more energy is released at lower values of friction, which is inconsistent with the former hypothesis. As mentioned above, fault slip velocity is higher during the interseismic period at lower normal stresses, and therefore, more acoustic energy is released prior to failure at lower normal stresses. Similarly, data from Experiment p5201 show that more acoustic energy is released prior to failure for higher shear velocities (Figures 4 and S3). Again, if acoustic energy tracked shear stress, we should expect to see more energy released at lower shear velocities. However, our data show that more energy released is at lower values of friction, which implies that slip velocity is the dominant factor in controlling the energy released prior to failure. These observations further confirm the results from our stable sliding data (Figure S1) and corroborate the idea that slip rate is the dominant effect on acoustic energy release (not shear stress).

To develop a more physical understanding behind the source of acoustic energy and to further verify that acoustic energy tracks slip velocity, we conducted conventional SHS tests and measured the amount of acoustic energy radiated before, during, and after the SHS (Figure 9a). Our data show that the acoustic energy tracks shear stress during the entire SHS test. At the onset of the hold, the acoustic energy immediately decreases and remains at a minimum for the duration of the hold. Upon reshear, the acoustic energy reaches a peak and then decays back to a steady‐state value (Figures 9a–9c). Since this entire process is analogous to the frictional behavior of the fault, we propose that the micromechanical processes that induce frictional healing are in fact the same processes that generate the release of acoustic energy. In particular, we propose that generation of acoustic energy is fundamentally related to the micromechanics of grain contact junctions. In terms of frictional healing, grain contacts are thought to increase in size and number due to chemical activated processes during the hold (Frye & Marone, 2002; Rabinowicz, 1951). As a result of this restrengthening process, the frictional strength increases upon reshear scales with duration of the hold time. We hypothesize that when the fault is locked (e.g., during the hold or linear‐elastic loading stage), the acoustic energy remains low because grain contacts are quasi‐stationary and growing in size and number. When the fault unlocks (e.g., during reshear of a SHS or inelastic loading), the acoustic energy begins to increase because grain contacts are being sheared and destroyed. This conceptual model is supported by both our SHS tests and our stick‐slip data sets.

### 4.2 The Effect of Grain Size and Contact Junction Size

Experiments conducted with different grain sizes demonstrate that grain size and, thus, contact junction size play a significant role in the temporal evolution and magnitude of acoustic energy release. Our data show that larger grain sizes produce more acoustic energy during the interseismic period and coseismic slip phase (Figures 11 and 12). For the largest grain size, the acoustic energy begins to increase well before failure and correlates with slip velocity (Figure 12a). However, as the grain size is reduced, the acoustic energy begins to increase later during the seismic cycle (Figures 12b and 12c). As mentioned above, slip velocity has a significant impact on the magnitude and temporal changes in elastic energy release. However, fault slip velocity alone cannot explain the acoustic energy trends in Figures 12b and 12c. That is, fault slip rate is highest during the interseismic period for fault zones composed of smaller grain sizes. Therefore, if slip velocity is the main control on acoustic energy release, we should expect to see an increase in energy released prior to failure for the smallest grain size. However, data in Figure 12 do not support this idea, and therefore, additional mechanisms must be considered.

Data presented in Figures 10, 11, 12 are conducted with the same initial layer thickness. However, since the median particle size is different for each experiment, the total number of grains across the gouge layer increases as particle size decreases. In particular, there are more grain contact junctions within a given volume (i.e., the particle coordination number) with decreasing grain size (Gheibi & Hedayat, 2018; Mair et al., 2002; Morgan & Boettcher, 1999). This implies that the true contact area per unit volume is higher for fault zones composed of smaller grain sizes. Furthermore, since the applied load is constant for each experiment, the average contact force on each particle is smaller for smaller grain sizes, due to a higher coordination number. Thus, if the average contact force decreases the shear strength of the material, stress drop and radiated acoustic energy should all decrease. This explanation is in good agreement with our data and is also consistent with previous works (Gheibi & Hedayat, 2018). This implies that in addition to fault slip velocity, the total number of contact junctions per unit volume (i.e., the true contact area) plays a key role in the generation of acoustic energy.

To conclude, our data show that in order for acoustic energy to be radiated, the total contact area per unit volume needs to be small (e.g., large grain sizes), and the fault needs to unlock and accelerate prior to failure. This finding could have important implications for the generation of microseismic activity and precursors to laboratory earthquakes and natural earthquakes. In particular, for the smallest grains studied, we did not detect microseismic precursors for laboratory earthquakes. This could imply that generation of foreshocks are controlled by fault zone maturity and/or fault zone comminution. However, additional work is needed, including utilizing active source ultrasonics and pore fluid pressure, to verify the role of particle coordination number and to explore implications of particle size for upscaling our results to mature faults zones.

### 4.3 ML and Prediction of Failure

The systematic evolution of acoustic energy throughout the seismic cycle is what ultimately enables accurate prediction of laboratory earthquakes. Here, we have begun to provide a physical basis for the ML‐based prediction using frictional contact mechanics. We find that the magnitude and the temporal evolution of radiated acoustic energy can be explained by changes in fault slip rate and the true contact area per unit volume within the fault zone. If our hypothesis is correct, then this implies that the ML‐based predictions of laboratory earthquakes are controlled by the breaking/sliding of contact junctions. Moreover, if the fault slip rate is low enough or if the total number of contact junctions per unit volume is large (e.g., small grain size), then there should be a lack of foreshocks and/or acoustic energy. A lack of AE activity would result in a decrease in the performance of the ML‐based predictions. This hypothesis is in part confirmed by Lubbers et al. (2018), who showed that the ML‐based predictions are closely related to the magnitude and frequency of foreshocks that occur before failure. However, more ML‐based studies are needed to verify if this hypothesis is indeed correct.

## 5 Conclusion

We analyze acoustic data from friction experiments for a range of boundary conditions and illuminate the physical processes that control the magnitude and temporal evolution of acoustic energy throughout the seismic cycle. Our data show that the magnitude of the acoustic energy released during coseismic failure scales with the stress drop of the slip event. We show that fault slip rate plays a key role in the generation of acoustic energy during the interseismic period. In addition, frictional contact area per unit fault volume dictates the magnitude and evolution of elastic radiation. Fault zones composed of smaller particles radiate less acoustic energy than fault zones composed of larger particles because the contact area per unit volume is higher for smaller grain sizes, and thus, the average contact forces exerted on each particle is smaller. We attribute the generation and evolution of acoustic energy to be fundamentally related to the microphysical processes acting at grain contact junctions. The magnitude of the acoustic energy is related to the real area of contact between neighboring grains, and the rupturing of grain contact junctions is one of the main physical mechanisms that generates the acoustic energy throughout the laboratory seismic cycle.

Our results have important implications for ML‐based prediction of microseismic activity and precursors to failure. Microseismic activity and precursors have a fundamental impact on the ability to improve earthquake early warning systems and possibly earthquake forecasting. Ultimately, our data suggest that generation of microseismic activity could be directly related to the fault slip rate and the true contact area per unit volume of the fault gouge. In the context of ML, our data show that the ML predictions are in some ways related to the slip rate of the fault. That is, the unlocking of the fault is a key parameter that dictates the temporal evolution of the acoustic energy. Future ML‐based studies should be devoted to understanding the effect of fault slip rate and grain contact size on the performance of ML models. More specifically, it remains unknown whether ML models can still predict the time to failure of impeding earthquakes if the fault remains locked and the generation of acoustic energy does not evolve throughout the seismic cycle.

## Supporting information

Supporting Information S1

## Data Availability

All data in this study are publicly available at https://scholarsphere.psu.edu (https://doi.org/10.26207/v5ha-5a25).
