# Supplementary material for: Acoustic Energy Release During the Laboratory Seismic Cycle: Insights on Laboratory Earthquake Precursors and Prediction
Source: J Geophys Res Solid Earth. 2020 Aug 11;125(8):e2019JB018975. doi: 10.1029/2019JB018975 (PMC7685124; doi:10.1029/2019JB018975)
Supplement: Supplementary file 1 — Supporting Information S1 [file JGRB-125-e2019JB018975-s001.docx]

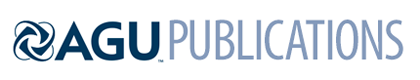


*Journal of Geophysical Research: Solid Earth*

Supporting Information for

**Acoustic Energy Release During the Laboratory Seismic Cycle: Insights on Laboratory Earthquake Precursors and Prediction**

David C. Bolton^1^, Srisharan Shreedharan^1^, Jacques Rivière^2^ and Chris Marone^1,3^

^1^ Department of Geosciences, Pennsylvania State University, University Park, Pennsylvania

^2^Department of Engineering Science and Mechanics, Pennsylvania State University, University Park, Pennsylvania

^3^Dipartimento di Scienze della Terra, La Sapienza Università di Roma, Italy

**Contents of this File:**

Figures S1-S3

**Introduction:**

This supporting information contains figures that demonstrate the effect of computing the acoustic variance (energy) using: (1) a constant time window, (2) a constant displacement window, or (3) a constant displacement window applied to decimated acoustic data. In addition, we show that acoustic energy is inversely related to shear stress.

In Figure S1, we plot acoustic variance and shear stress for data at different shearing velocities from Experiment p5348. In this particular experiment, we do *not* use any acrylic spring in series with the vertical ram, which promotes stable frictional sliding. Variance is computed using a constant displacement window of 5 µm in S1A. Therefore, the length of each moving window in time (N in Equation 1) changes systematically with shearing velocity. More specifically, the window size becomes larger in time with decreasing shear velocity. The data show that more energy is radiated at higher shear velocities. Since acoustic variance is normalized by N, one could argue that the results in Figure S1A are simply due to the effect of N and a smaller window size relative to the data at lower shear velocities. To verify that the results in Figure S1A are independent of the window size (N), we compute variance using a 5 µm window for each shear velocity and we decimate the acoustic data such that N is the same for each velocity (Figure S1B). More specifically, N is smallest at 60 µm/s so the acoustic data corresponding shear velocities <60 µm/s are decimated such that each moving window of 5 µm contains the same number of data points (N) as the 60 µm/s case. The values of variance are the exact same as those shown in Figure S1A. Therefore, we can conclude that the size of the window (N) has no effect on energy radiation during stable sliding. In addition, we verify that energy radiation is independent of slip displacement by computing variance using a constant time window of .1s in Figure S1C. Again, the acoustic variance increases with shear velocity and the absolute values of variance are approximately the same as the variance in Figure S1A. Since the absolute values of variance are the same in Figures S1A and S1C, this implies that acoustic energy radiation is independent of slip displacement. In Figure S2, we plot variance as a function of time for two different shear velocities from Experiment p5201. The data demonstrate that the inter-seismic changes in variance are independent of window length. However, the peak energy radiated during co-seismic slip changes systematically with window length (Figure S2). Thus, using different window sizes can influence the co-seismic trends, but does not affect the inter-seismic trends. This implies that a constant window in time is the correct way to compute energy because N remains constant. Furthermore, to avoid windowing effects associated with the energy release during co-seismic slip, we report all of our co-seismic data in terms of the cumulative energy release instead of peak energy (see main text for details). Lastly, we demonstrate that the energy released during the inelastic loading phase is not correlated with friction (Figure S3). Our data clearly show that more energy is released at lower values of friction during the inter-seismic period. This implies, that energy released during the inelastic loading phase is more correlated with slip rate than stress.

**Figure S1.** Three methods to calculate acoustic signal variance for different shearing velocities, all plotted along with shear stress during stable sliding. **A**. Acoustic variance for a time window corresponding to a shear displacement of 5 µm; thus a factor of 30 longer window for 2 µm/s compared to 60 µm/s. Note that variance increases with shear velocity. **B.** Same as Panel **A** except the acoustic data are decimated such that the number of data points per window is the same for each velocity. Note that variance is identical to Panel **A**. **C.** Variance computed using a constant time window of .1s. The absolute values of variance are approximately the same as for **A** and **B** indicating that the amount of energy radiated is independent of slip displacement.

**Figure S2. A-B.** Variance plotted as a function of time at two different shearing velocities from Experiment p5201 (see main text for details). Variance is computed using two different window sizes (black and red). The data in black correspond to constant displacement window of 5 µm. The inter-seismic changes in variance are independent of window size, but the co-seismic peaks change systematically with window length.

**S3. A.** Variance versus friction for data corresponding to the onset of inelastic creep until peak shear stress (see main text for details) from Experiment p5198. The data show that more energy is released prior to failure for lower normal stresses. **B.** Same as A, but data here correspond to Experiment p5201. Similar to A, more energy is released prior to failure for higher shear velocities.
